# Do clinical caries-preventive interventions applied to expectant mothers affect caries-related parameters in their children? A systematic review

**DOI:** 10.1007/s40368-025-01025-6

**Published:** 2025-04-09

**Authors:** Farida Abdunabi, Mawlood Kowash, Miltiadis A. Makrygiannakis, Eleftherios G. Kaklamanos

**Affiliations:** 1 Hamdan Bin Mohammed College of Dental Medicine, Mohammed Bin Rashid University of Medicine and Health Sciences, Dubai, United Arab Emirates; 2 School of Dentistry, National and Kapodistrian University of Athens, Athens, Greece; 3 School of Dentistry, European University Cyprus, Nicosia, Nicosia Cyprus; 4 School of Dentistry, Aristotle University of Thessaloniki, Thessaloniki, Greece

**Keywords:** Early childhood caries, Intervention, Pregnancy, Prevention, *Streptococcus mutans*

## Abstract

**Purpose:**

To assess whether caries-preventive clinical interventions targeted exclusively to expectant mothers reduce caries experience or various surrogate parameters associated with caries activity in their children.

**Method:**

Seven databases were searched for randomised clinical trials until November 2024. Studies assessing clinical caries-preventive measures in healthy expectant mothers and comparing them to placebo, other, or no interventions. Outcomes measured in children included caries experience and surrogate parameters (e.g., *S. mutans* levels), patient-reported, and socioeconomic data. Non-randomized studies as well as, behavioural and educational interventions were excluded. Risk of bias was assessed using the Cochrane Collaboration’s RoB 2 tool.

**Results:**

Out of the 2149 studies identified, only 5 studies were included. Two of them presented with low risk of bias, whereas there were some concerns for the other three. The assessed outcomes included either caries experience directly (percentage of caries active children, DMFT, DMFS, dmft, dmfs) or the *Streptococcus mutans* levels. Children in the experimental groups showed significantly lower *S. mutans* levels compared to controls. Regarding caries levels, the results were contradictory. The supplementation of calcium in expectant mothers resulted in caries reductions in their offsprings, while sodium fluoride with potassium iodine topical applications or vitamin D supplementation were not shown to be effective.

**Conclusion:**

Clinical caries-preventive interventions applied to expectant mothers might result in reduced *S. mutans* levels in their children. Further studies are warranted to provide additional evidence, especially with regards to caries experience.

**Registration:**

Research Registry: reviewregistry1047.

**Supplementary Information:**

The online version contains supplementary material available at 10.1007/s40368-025-01025-6.

## Introduction

Dental caries is one of the most widespread and prevalent noncommunicable childhood diseases worldwide. Its aetiology is multifactorial, diet-modulated, biofilm-mediated, and dynamic (MacHiulskiene et al. 2020), resulting in a net mineral loss of dental hard tissues. Early childhood caries (ECC) is a progressive, irreversible (if cavitated) microbial disease affecting the hard tissues of the tooth. It is the most common chronic human disease and can result in pain, infection, and impaired nutrition and overall health. Additionally, ECC is expensive to treat, placing a significant burden on healthcare systems (Agili 2013).

The prevalence of ECC varies significantly among countries. In Western countries such as Sweden, the prevalence is relatively low, accounting for 11.4% among 3–6-year-olds, particularly in disadvantaged groups (Anil and Anand 2017). In contrast, higher prevalence rates have been reported in Middle Eastern countries. For example, in the United Arab Emirates (UAE), the prevalence of ECC ranges from 74.1% to 83% in 4–5-year-olds, with a decayed, missing, and filled teeth index (dmft) ranging from 3.07 to 10.9 (Al Ayyan et al. 2018; Kowash et al. 2017; Kowash 2015). Similar trends have been observed for caries in permanent dentition. For instance, the Child Dental Health Survey 2013 in England, Wales, and Northern Ireland reported caries prevalence rates of 34% and 40% in 12- and 15-year-olds, respectively (Holmes et al. 2013). In the Gulf Cooperation Council (GCC) countries, a recent systematic review revealed an overall mean decayed, missing, and filled teeth (DMFT) index of 2.57 in permanent teeth, with a caries prevalence of 64.7% (Al Ayyan et al. 2018).

Early childhood caries has been defined as “the presence of one or more decayed (non-cavitated or cavitated lesions), missing (due to caries), or filled tooth surfaces in any primary tooth in a child 71 months of age or younger” (Drury et al. 1999). The infection typically begins in the maxillary central incisors and rapidly progresses to the remaining primary teeth (Aljarallah et al. 2018). ECC is considered a significant public health threat, being five to eight times more common than asthma (Alantali et al. 2020) and seven times more common than hay fever (Begzati et al. 2015). Wyne (1999) classified ECC into three categories based on severity: Type I (mild to moderate), characterized by isolated carious lesions affecting molars and/or incisors; Type II (moderate to severe), involving labio-lingual carious lesions in maxillary incisors, with or without molar caries; and Type III (severe), where carious lesions affect all teeth, including the lower incisors (rampant caries).

ECC is a complex multifactorial disease caused by the interaction of multiple biological, behavioural, and environmental factors. The primary causative factors include cariogenic microorganisms, fermentable carbohydrates, and a susceptible tooth surface or host (Anil and Anand 2017; Begzati et al. 2015). Additional risk factors include socioeconomic status, parental education, dietary habits, environmental influences, and pre-existing systemic diseases (Ezer et al. 2010). A recent systematic review and meta-analysis conducted by Bahardoust et al. (2024) reported that lower prenatal vitamin D levels are associated with a higher risk of dental caries compared to higher levels. Maintaining adequate vitamin D throughout pregnancy is essential as a preventive measure. Deficiency in late pregnancy (> 24 weeks) or the third trimester may further increase the risk. However, due to study variability and potential influencing factors, further research is needed to confirm these findings across diverse populations.

*Streptococcus mutans* levels have been strongly correlated with caries development in children (Çolak et al. 2013), with higher levels leading to increased decay rates (Anil and Anand 2017; Begzati et al. 2015; Tinanoff and O’Sullivan 1997). Vertical transmission of *S. mutans* from mother to child, often through saliva, is a significant route of infection (Harris et al. 2004; Reisine et al. 2008). Reducing *S. mutans* levels in parents, particularly mothers, has been shown to lower the risk of ECC in children (Begzati et al. 2015; Ismail 1998).

Behavioral factors, such as bottle-feeding at night, also play a critical role in ECC development (Kowash 2015). Socioeconomic factors further exacerbate the risk, often due to limited access to resources and a lack of education (Folayan et al. 2020, 2024; AAPD 2024).

Recently, Saxena et al. (2024) conducted a systematic review and meta-analysis suggesting that educating mothers about oral health might help prevent ECC in their children. However, the strength of this recommendation remains weak, largely due to the variability in intervention methods and the limited number of studies on the topic.

Several studies have suggested that professional interventions during the prenatal period may reduce the incidence of ECC (Begzati et al. 2015; Xiao et al. 2019; Leverett et al. 1997; Schroth et al. 2014).

This systematic review aims to summarize the available scientific evidence from randomized clinical trials assessing the effectiveness of caries-preventive clinical interventions targeted exclusively to expectant mothers regarding the reduction of caries experience or various surrogate parameters associated with caries activity (e.g., *S. mutans* presence, *S. mutans* levels, etc.), in their children. The null hypothesis is that there is no difference in the reduction of caries experience or the various surrogate parameters associated with caries activity, in the children of expectant mothers receiving caries-preventive clinical interventions compared to placebo, other interventions or no intervention.

## Materials and methods

### Protocol and registration

At the beginning a special protocol was developed and piloted (registration in Research Registry: reviewregistry1047). Relevant methodological guidelines were followed regarding conduct and reporting (Beller et al. 2013; Higgins et al. 2024; Page et al. 2021; Shamseer et al. 2015). As the present study was a systematic review, ethical approval was not required.

### Research question

The research question of the present review is the following:

Do caries-preventive clinical interventions targeted exclusively to expectant mothers reduce caries experience or various surrogate parameters associated with caries activity in their children?

### Eligibility criteria

The eligibility criteria were defined based on the Participants, Intervention, Comparison conditions, Outcomes and Study design domains.

#### Types of participants

Pregnant women were the participants in this review.

#### Types of interventions

Clinical caries-preventive measures targeted exclusively to expectant mothers involving the supplementation of chemical or antimicrobial agents like fluoride, calcium, vitamins, xylitol, etc. The interventions could be continued post-partum.

#### Types of comparison conditions

Placebo, other interventions, standard intervention, or no intervention.

#### Types of outcome measures

The primary outcomes included either direct caries experience (e.g., percentage of caries-active children, DMFT, DMFS, dmft, dmfs, etc.) or various surrogate parameters associated with caries activity (e.g., *S. mutans* presence, *S. mutans* levels, etc.), in their children, of both genders and at various ages post-partum. Secondary outcomes included patient-reported outcomes and socioeconomical assessments.

#### Types of studies

Randomised controlled trials (RCTs), published or unpublished, as well as abstracts of unpublished studies, were deemed eligible for inclusion.

We excluded the following types of studies: investigation of behavioural or educational interventions, non-randomized studies and non-comparative studies (case reports and case series). Also, we excluded animal studies; ex vivo, in vitro, in silico studies; reviews (traditional reviews, systematic reviews and meta-analyses) (Supplementary Table 1).

### Information sources and search strategy

Following the development of detailed search strategies by EGK, the other authors searched the whole content in 7 electronic databases from inception until November 2024 (PubMed, Cochrane Central Register of Controlled Trials (CENTRAL), Cochrane Database of Systematic Reviews, SCOPUS, Web of Science, ProQuest Dissertations and Theses Global, Google Scholar) (Supplementary Table 2). ProQuest Dissertations and Theses Global database, Google Scholar, as well as ClinicalTrials.gov and the WHO's International Clinical Trials Registry Platform accessed through CENTRAL were searched in order to retrieve unpublished literature. The searches were conducted without placing restrictions on English language and were supplemented by reviewing the bibliography in any relevant paper retrieved and manual searches. Moreover, we had planned to contact the corresponding author in the event we needed some clarifications on the content of a potentially eligible paper.

### Study selection, data collection and data items

The investigators (FA, MK and MAM) assessed the retrieved records for inclusion separately without being blinded about the identity of the authors and kept a record on all decisions. Kappa statistics were not computed following relevant recommendations (Higgins et al. 2024). They also assessed, again independently, the full report of records considered by either reviewer to meet the inclusion criteria. From the finally included studies, data extraction was carried out by filling in special pre-piloted forms the following items: bibliographic data; information on study design; participants’ eligibility criteria and characteristics; details on the interventions studied; outcomes measurement and results.

### Risk of bias in individual studies

Two authors (FA and EGK) assessed the risk of bias in individual studies, independently and in duplicate, using The Cochrane Collaboration’s RoB 2 tool (Higgins et al. 2024). Any disagreements in the processes described above, were resolved by discussion.

### Summary measures and synthesis of results

If deemed possible, the outcomes reflecting caries experience, and the surrogate microbiological parameters related to caries were planned to be expressed as Standardized Mean Differences (WMD) together with a 95% Confidence Interval (CI) (Higgins et al. 2024).

The random effects method for meta-analysis were to be used to combine data (Borenstein et al. 2009; DerSimonian and Laird 1986), since they were expected to differ across studies due to diversity in terms of subject groups, procedures and follow-up. To identify the presence and extent of between-study heterogeneity, an overlap of the 95% CI for the results of individual studies was to be inspected graphically and the I^2^ statistic was to be calculated (Higgins et al. 2024).

All analyses were to be done with Comprehensive Meta-analysis software 2.2.046 (©2007 Biostat Inc.). Significance (a) was to be set at 0.05, except for the 0.10 used for the heterogeneity tests (Ioannidis 2008).

### Risk of bias across studies and additional analyses

If a sufficient number of studies were identified, analyses were planned for “small-study effects” and publication bias (Higgins et al. 2024). If deemed possible, exploratory subgroup analyses were planned according to intervention characteristics. In addition, the quality of evidence was planned to be assessed based on the Grades of Recommendation, Assessment, Development and Evaluation (GRADE) approach (Guyatt et al. 2011).

## Results

### Study selection

**Fig. 1:**
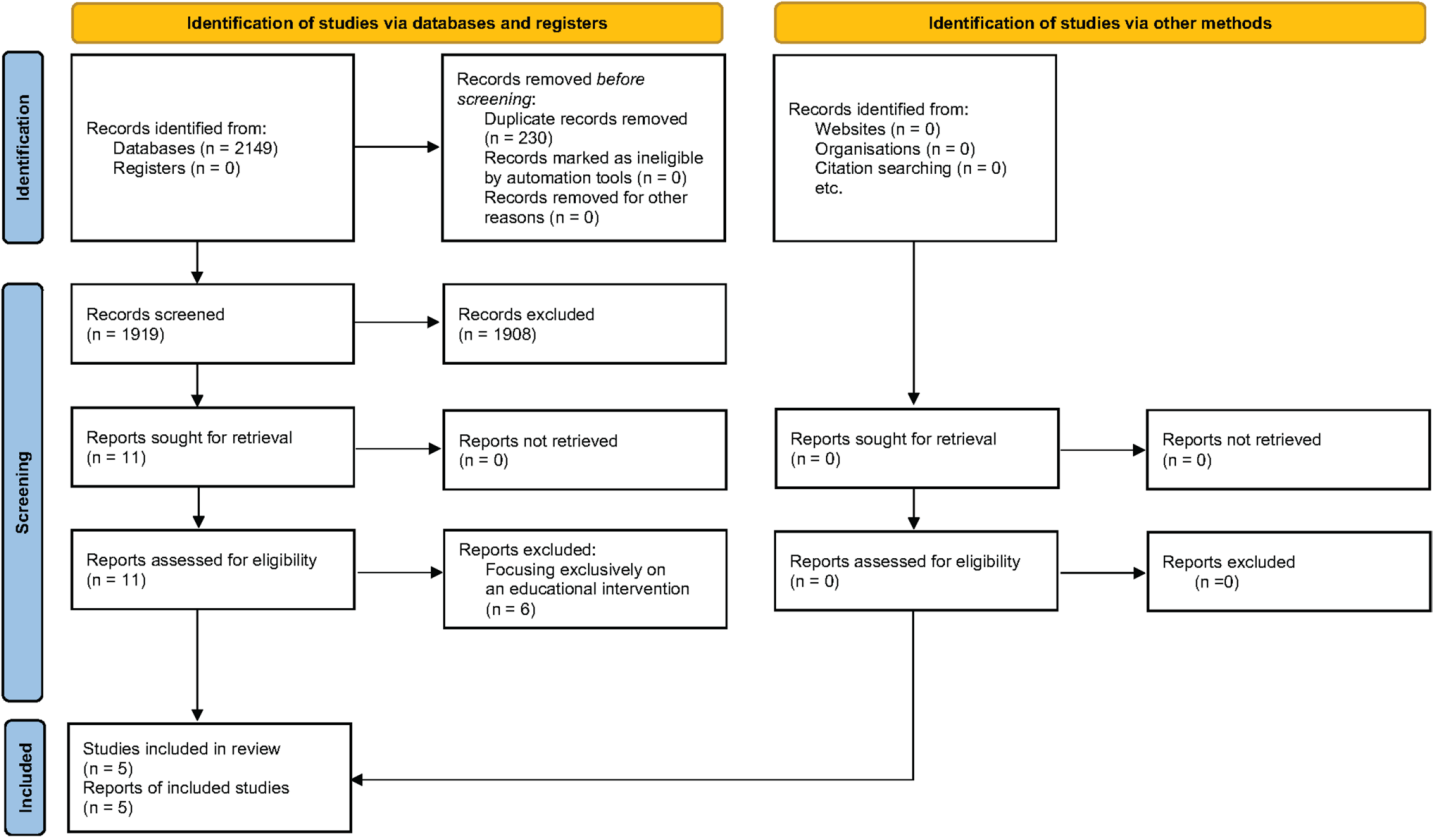
Flowchart of records

From the 2149 initially identified records, we excluded 230 as duplicates, 1908 more based on their title and abstract and 6 after reading the full text as they focused exclusively on an educational intervention(Birungi et al. 2015; Harrison et al. 2012; Lucey 2009; Plutzer et al. 2012; Plutzer 2010; Plutzer and Spencer 2008). Finally, 5 full-text reports were included in the systematic review (Bergel et al. 2010; Brambilla et al. 1998; Nakai et al. 2010; Nørrisgaard et al. 2019; Zanata et al. 2003) (Fig. 1).

### Study characteristics

The characteristics of the included studies are presented in Tables 1 and 2. The trials had been published from 1998 to 2019 and involved various interventions in groups of pregnant women after the 2nd trimester of pregnancy, like calcium tablet and Vitamin D supplementation (Bergel et al. 2010; Nørrisgaard et al. 2019), xylitol gum (Nakai et al. 2010), sodium fluoride/chlorhexidine rinses (Brambilla et al. 1998) and sodium fluoride/potassium iodine topical application (Zanata et al. 2003). Two studies included women with salivary *S. mutans* levels > 10^5^ CFU/ml (Brambilla et al. 1998; Nakai et al. 2010). The follow-up period lasted from 2 years (Nakai et al. 2010) up to 12 years (Bergel et al. 2010). The latter constituted a follow-up of a cohort of children, who were originally enrolled in a randomized clinical trial of mother calcium supplementation to prevent hypertensive disorders of pregnancy (Bergel et al. 2010). In the located studies the investigators assessed either directly caries experience (percentage of caries active children, DMFS, dmft) (Bergel et al. 2010; Nørrisgaard et al. 2019; Zanata et al. 2003) or the *S. mutans* levels (saliva or plaque) (Brambilla et al. 1998; Nakai et al. 2010). No studies assessing person-reported outcomes or socioeconomic evaluations were retrieved. Sample size had been calculated in advance in all but one study (Brambilla et al. 1998), while compliance was monitored by Bergel et al. 2010, Nørrisgaard et al. 2019 and Nakai et al. 2010.

**Table 1:** General characteristics of the studies included in the systematic review and main results

Study	Intervention characteristics	Outcomes [differences between groups; p value]*			Additional information
Bergel et al. 2010 [Argentina]	EG: Calcium 2g; daily from 20th week of gestation to delivery; per os CG: Placebo; daily from 20th week of gestation to delivery; per os	Assessed at 12 years Proportion of caries-active children (permanent/primary dentition): EG < CG [< 0.001] Proportion of caries-active children (permanent dentition): EG < CG [< 0.001] Proportion of caries-active children (primary dentition): EG < CG [0.503] DMFS + dmfs: EG < CG [< 0.001]			A priori sample calculation: Yes Monitoring of compliance: Yes
Brambilla et al. 1998 [Italy]	EG: Sodium fluoride 0.05%/ chlorhexidine 0.012%; daily rinses; 3 cycles of 20 days each, with two 10-day rinse-free intervals (starting from the end of the 6th month of pregnancy) CG: No placebo given Both groups received: Dietary counselling; professional prophylaxis; 1 mg systemic fluoride daily (starting from the end of the 6th month of pregnancy)	Assessed at 6, 12, 18 and 24 m *Streptococcus mutans* levels (saliva) 06 m: EG < CG [> 0.05] 12 m: EG > CG [> 0.05] 18 m: EG < CG [< 0.05] 24 m: EG < CG [< 0.01]			A priori sample calculation: NR Monitoring of compliance: NR
Nakai et al. 2010 [Japan]	EG: Xylitol; 1 gum pellet containing 1.32 g to be chewed at least 4 times a day; started at the 6th month of pregnancy and terminated 13 months later, when the children were 9 months old CG: No placebo given Both groups received: Basic preventive program (oral hygiene instructions and professional tooth cleaning at 6th month of pregnancy)	Assessed at 6, 9, 12, 18 and 24 m			A priori sample calculation: Yes Monitoring of compliance: Yes
		*Streptococcus mutans* levels (saliva from tongue)	*Streptococcus mutans* levels (saliva from gingiva or plaque from tooth surfaces)^§^	± in combined sites	
		06 m: EG < CG [0.412] 09 m: EG < CG [0.014] 12 m: EG < CG [< 0.001] 18 m: EG < CG [0.121] 24 m: EG < CG [0.007]	06 m: EG < CG [0.167] 09 m: EG < CG [0.003] 12 m: EG < CG [< 0.001] 18 m: EG < CG [0.216] 24 m: EG < CG [0.014]	06 m: EG < CG [0.060] 09 m: EG < CG [< 0.001] 12 m: EG < CG [< 0.001] 18 m: EG < CG [0.021] 24 m: EG < CG [0.035]	
Nørrisgaard et al. 2019 [Denmark]	EG: High dose Vitamin D3; 2400 IU/d; from 24th week of gestation to 1 w post-partum CG: Matching placebo tablets; from 24th week of gestation to 1 w post-partum Both groups received: 400IU/d Vitamin D3 in addition to 2.4 g/day of long-chain ω-3 polyunsaturated fatty acids (PUFAs) during pregnancy	Assessed at 6y Proportion of caries-active children Permanent dentition: EG > CG [> 0.05] Primary dentition: EG < CG [> 0.05} Permanent and/or primary dentition: EG > CG [> 0.05]			A priori sample calculation: Yes Monitoring of compliance: Yes
Zanata et al. 2003 [Brazil]	EG: Sodium fluoride 1.2%/potassium iodine 1%; topical application; administered in 3 sessions (the first immediately after prophylaxis, and the second and third applications after 3 and 5 days); elimination of infection sites through tooth extraction, endodontic dressings, root scaling and sealing of cavities; Sodium fluoride/potassium iodine applied at 6 and 12 m post-delivery also CG: No placebo given; posterior teeth cavities filled with zinc oxide-eugenol cement; anterior teeth cavities filled with composite Both groups received: Preventive and educational program	Assessed at 24 m Proportion of caries-active children (permanent/primary dentition): EG < CG [0.080]			A priori sample calculation: Yes Monitoring of compliance: NR

CG: Control Group; DMFS; decayed, missing, filled surfaces in the permanent dentition; dmfs: decayed, missing, filled surfaces in the primary dentition; EG: Experimental Group; g: gram(s); m: month(s); NR: not reported; y: year(s); ± : presence/absence

^*^Statistically significant differences in bold

^§^ *Streptococcus mutans* levels in unstimulated saliva samples from mucosa of mandibular and maxillary ridges when a tooth was absent, or in plaque samples from tooth surfaces when they were present

**Table 2:** Participant characteristics of the studies included in the systematic review

Study	Eligibility criteria for the included mothers	Mothers randomized	Children analyzed^§^
Bergel et al. 2010 [Argentina]	Inclusion criteria: nulliparous; singleton pregnancy; less than 20 weeks pregnant; blood pressure < 140/90 mmHg Exclusion criteria: clinical or laboratory evidence of present or past disease; taking medication; abnormal glucose-tolerance	Experimental Group: 593 Control Group: 601	Experimental Group: 98 Control Group: 97
Brambilla et al. 1998 [Italy]	Inclusion criteria: last week of the 3rd month of pregnancy; salivary *Streptococcus mutans* levels > 10^5^ CFU/ml Exclusion criteria: NR	Experimental Group: 33 Control Group: 32	Experimental Group: 31 Control Group: 29
Nakai et al. 2010 [Japan]	Inclusion criteria: 3rd to 5th month of pregnancy; salivary *Streptococcus mutans* levels > 10^5^ CFU/ml Exclusion criteria: antibiotic use during the prior month; gastrointestinal problems	Experimental Group: 56 Control Group: 51	Experimental Group: 46 Control Group: 31
Nørrisgaard et al. 2019 [Denmark]	Inclusion criteria: 24th week of pregnancy Exclusion criteria: any endocrine, cardiovascular, or nephrologic disorders or vitamin D3 (cholecalciferol) intake greater than 600 IU/d	Experimental Group: 315 Control Group: 308	Experimental Group: 244 Control Group: 252
Zanata et al. 2003 [Brazil]	Inclusion criteria: 2nd or 3rd third trimester of pregnancy; without any medical contraindications that could make dental treatment inadvisable; presenting three or more active carious lesions (cavities) in smooth dental surfaces Exclusion criteria: NR	Experimental Group: 43 Control Group: 38	Experimental Group: 34 Control Group: 30

*CFU* colony-forming units, *NR* not reported

^§^at the longest follow-up available

### Risk of bias within studies

**Fig. 2:**
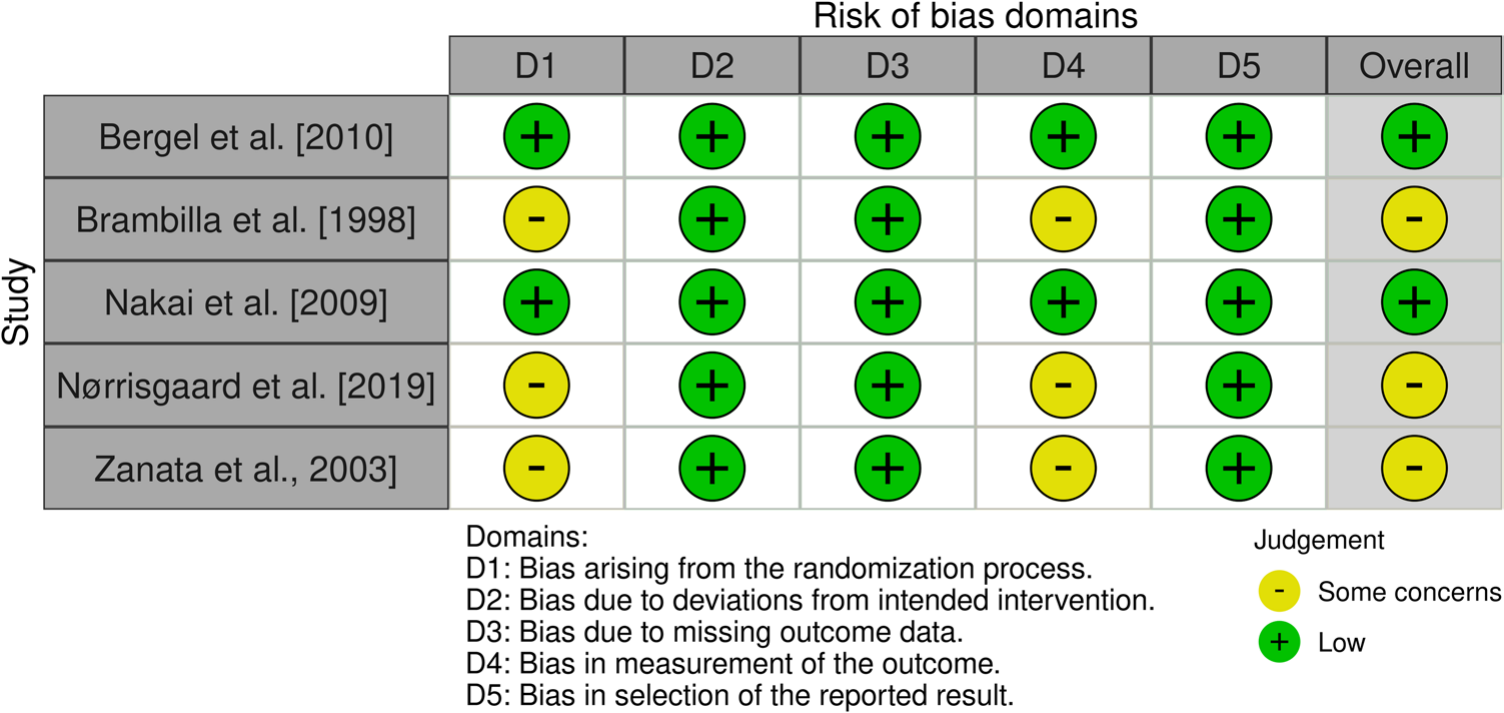
Risk of bias assessment with the Cochrane Collaboration's RoB2 tool

Figure 2 presents the findings of the risk of bias assessment. Two studies were overall assessed to be at a low risk of bias (Bergel et al. 2010; Nakai et al. 2010), whilst there were some concerns for the other three publications: Brambilla et al. (1998), Nørrisgaard et al. (2019) and Zanata et al. (2003).

Bergel et al. (2010) and Nakai et al. (2010) investigations were assessed at a low risk of bias regarding all domains. Although both studies reported high attrition rates, there is no evidence that missing data affected the outcome, thus the risk of bias was considered to be low. Nakai et al. did not use a placebo intervention but given the specific study design we do not expect this to have affected bias. Brambilla et al. (1998), Nørrisgaard et al. (2019) and Zanata et al. (2003) were found to exhibit some concerns arising from the randomization process associated with uncertainties regarding random sequence generation and allocation concealment. Moreover, there were some concerns regarding the measurement of the outcome because of the processes relevant to the blinding of the assessments. No study used a placebo intervention except Nørrisgaard et al. (2019); however, similarly to the rationale previously explained we do not expect this to have affected bias. Zanata et al. (2003) exhibited a quite high attrition rate but again there is no evidence that these attrition rates affected the outcome.

### Study findings

#### Caries experience

Three of the included studies assessed caries experience directly by investigating the percentage of children with active caries, DMFS and dmft following various interventions (Table 1) (Bergel et al. 2010; Nørrisgaard et al. 2019; Zanata et al. 2003).

Bergel et al. observed that following supplementing pregnant mothers with 2g calcium daily from the 20th week of gestation until delivery, the proportion of caries-active children in both permanent and primary dentitions (Experimental Group (EG): 63.3%; Control Group (CG): 86.6%; *p* < 0.001), as well as the sum of DMFS and dmfs (EG (mean ± standard deviation): 3.1 ± 4.05; CG: 4.4 ± 4.11; *p* < 0.001), was less in the 12 year-old children of the experimental group compared to those receiving the placebo tablets. The same was true for the differences in caries experience in the permanent dentition (EG: 60.2%; CG: 81.4%; *p* < 0.001). However, no difference was noted when the presence of caries was considered in the primary dentition separately (EG: 11.2%; CG: 14.4%; *p* = 0.503).

On the contrary, no difference was noted in the proportion of caries active children after the administration to pregnant mothers of sodium fluoride plus potassium iodine topical application (2 year-olds—primary dentition; EG: 14.7%; CG: 33.3%; *p* = 0.08; Zanata et al. 2003) and increased Vitamin D dosage (6 year-olds—primary and permanent dentition; EG: 22.1%; CG: 21.0%; *p* > 0.05; Nørrisgaard et al. 2019), respectively.

#### S. mutans levels

The other two located studies investigated the offspring’s colonization with *S. mutans* after using daily rinses or chewing xylitol gums (Table 1) (Brambilla et al. 1998; Nakai et al. 2010).

Brambilla et al. showed that children from mothers participating in the experimental group (rinses with sodium fluoride and chlorhexidine) showed significantly lower *S. mutans* levels compared to the control group (18 months post-partum, *p* < 0.05; 24 months post-partum, *p* < 0.01). A total of 24 out of the 29 control group children (83%) became permanently infected with *S. mutans* (concentration greater than 10^3^ CFU/ml), while in the experimental group, this occurred in 15 out of 31 children (48%) (*p* < 0.01). Additionally, more children in the control group exhibited *S. mutans* levels at above the risk threshold level of 10^5^ CFU/ml, compared to the experimental group (EG:46%; CG:95%; *p* < 0.01). The mean age at which colonization occurred in EG was 22.53 months, compared with 18.20 months for children whose mothers received no intervention (*p* = 0.003).

Similarly, Nakai et al. (2010) found that the daily use of xylitol gums by the expectant mothers can significantly reduce the overall prevalence of colonization in their children 2 years after delivery (EG: 63%; CG: 87.1%; *p* = 0.035). The same was also observed for *S. mutans* levels in saliva from tongue (*p* = 0.007) or saliva from the gingiva or plaque from surfaces of teeth (*p* = 0.014).

### Summary measures, synthesis of results, risk of bias across studies and additional analyses

Due to the methodological heterogeneity observed between the retrieved studies, it was not possible to conduct a meta-analysis. Similarly, subgroup analyses, analyses for “small-study effects” and publication bias, as well as the assessment of the quality of evidence following Guyatt et al. (2011) were planned but not finally performed due to the inadequate number of retrieved studies (Higgins et al. 2024).

## Discussion

### Summary of evidence

The systematic review aimed to evaluate the effectiveness of interventions aimed at expectant mothers on reducing caries-related parameters in their children. Overall, the studies investigating offspring’s colonization with *S. mutans* showed evidence in support to the intervention used (Brambilla et al. 1998; Nakai et al. 2010). Regarding caries levels the results were contradictory. Bergel et al. (2010) showed less caries in the intervention group, while Zanata et al. (2003) and Nørrisgaard et al. (2019) did not detect statistically significant differences.

The window of infectivity was described by Caufield et al. in 1993 and was described as the initial acquisition of *S. mutans* from the maternal host (Caufield et al. 1993). The window spans from 19 months until 31 months of age, the median age being 26 months. The clinical importance of the window of infectivity allows us to understand that the earlier the child acquires the cariogenic bacteria from the mother, the higher is the child's susceptibility to childhood caries later in their childhood (Assiry 2018). Nakai et al. (2010) and Brambilla et al. (1998) assessed the *S. mutans* levels in children at 24 months. Both studies were conducted during the window of infectivity, allowing us to speculate that children with lower *S. mutans* levels during this window are less likely to develop caries later in their childhood. While the window of infectivity is described to occur between 19 and 31 months, the peak onset of ECC in children is 3–4 years of age (Xiao et al. 2019). Excluding the 12-year study by Bergel and co-workers, the retrieved studies did not assess the intervention's efficacy during this time frame. Studies extending during the peak onset age would further reinforce the association between the intervention given and its efficacy in reducing ECC.

Prevention remains certainly the solution for the continuing problem of ECC. In this context, there are two main preventive programs: individual-based intervention and community-based intervention (American Academy of Pediatric Dentistry (AAPD) 2012). Individual-based intervention options are consistently used for high-risk populations, as they are easy to apply and do not require much effort from the parent or caregiver. However, it might be expensive as it is performed by professional or auxiliary personnel. Preventive interventions can be addressed either the mother or child (American Academy of Pediatric Dentistry (AAPD) 2012; Köhler et al. 1983; Lee et al. 1994). In the case of the mother, this can be accomplished by either counselling, tooth brushing and/or use of fluoride to reduce the number of maternal S. mutans. Meanwhile, in children, the approach is through the application of fluoride varnish and xylitol. Community-based prevention requires collaborations among parents or caregivers, health professionals and the community which are decisive to solve the problem of ECC efficiently and possibly other diseases related to poor nutrition. Marriott and co-workers reported that in young children an inappropriate diet may not only be contributing to poor oral health, but also to develop a poor nutritional outcome (Marriott et al. 2010). It has been reported that generally the community dental health programs are more effective than individual-based preventive approaches (Weintraub 1998). However, to be successful and effective, the community-based options should consider the social belief and culture as well as the level of education of parents (Mattheus 2015).

Moreover, there are a few points to be taken into consideration in the interpretation of the located results. One point that must be examined relates to the low number of studies obtained after study selection. Of the five studies, three had caries risk reduction as their primary aim (Brambilla et al. 1998; Nakai et al. 2010; Zanata et al. 2003). Meanwhile, the fourth aimed to primarily assess enamel defects in offspring of mothers consuming increased quantity of Vitamin D (Nørrisgaard et al. 2019) and the fifth had as a goal to assess the possible association between calcium intake prenatally and hypertension (Bergel et al. 2010). Dental caries was a secondary outcome of both studies. The limited number of studies precludes broader interpretations and conclusions and renders further investigations warranted.

Another point to consider is the variation in intervention modalities. Two studies assessed fluoride supplementation (Brambilla et al. 1998; Zanata et al. 2003). Optimal exposure to fluoride is important to all dentate infants and children (Garcia et al. 2001). The included studies employed different concentrations. Brambilla et al. (1998) used a 0.05% sodium fluoride mouth rinse, while Zanata et al. (2003) used a 1.2% concentration. Zanata et al. (2003) used simultaneously an iodine solution, while Brambilla et al. (1998) a chlorhexidine solution.

Apart from fluoride, xylitol (Nakai et al. 2010), calcium (Bergel et al. 2010) and Vitamin D supplements (Nørrisgaard et al. 2019) were investigated. Finally, the control groups of all five studies received different basic programs. This variation makes it challenging to come to a sound conclusion on which intervention method is most effective when compared to another. Furthermore, the assessment tools varied amongst the included studies. Using the same assessment tool for the different intervention methods would allow for more direct comparisons.

On a different note, it is important to understand the effect interventions on mothers, as well. Brambilla et al. (1998) and Zanata et al. (2003) described the effect on mothers as being similar to that of children. Sodium fluoride and chlorhexidine intervention to significantly reduce the *S. mutans* levels of mothers by six times (Brambilla et al. 1998). Mothers of children with high caries activity, exhibited higher caries levels than mothers of children with low caries levels (Zanata et al. 2003). The other located studies did not report the effect on the maternal dentition. Kowash observed that dental health education through home visits to mothers with young children significantly improved oral health in children and oral hygiene practices in mothers (Kowash 2015).

Xiao et al. (2019) examined oral health care interventions to pregnant women and infants regarding ECC prevention. In contrast, our review specifically evaluated clinical caries-preventive interventions (e.g., fluoride, xylitol, calcium, and vitamin D supplementation) administered exclusively to expectant mothers. By eliminating the confounding factor of parallel interventions for children, our review allows for a more precise assessment of clinical strategies' effectiveness in reducing caries-related outcomes. Moreover, our review explicitly excludes behavioral and educational interventions, ensuring that clinicians and policymakers receive targeted evidence on clinical approaches for ECC prevention. Although no new primary studies have been published since Xiao et al. (2019), the present systematic review included a more recent search (up to November 2024) from seven online databases to extract data and applied stricter inclusion criteria, limiting the selection to randomized controlled trials (RCTs) only. Notably, our review identified and included four additional RCT studies that were not covered previously, thereby strengthening the evidence base. Finally, the most recent Cochrane Collaboration’s RoB 2 tool was used to assess the risk of bias of the included studies.

### Limitations

Whilst presented with strengths, the present systematic review has some limitations. One of the limitations include the concerns of bias detected during the application of the ROB 2 assessment tool in three of the included studies (Brambilla et al. 1998; Nørrisgaard et al. 2019; Zanata et al. 2003). Such concerns might curtail the reliability of the data in the assessment of interventions effectiveness.

### Recommendations for future studies

Additional studies are required to fully assess the efficacy of the different experimental protocols with a larger number of participants. New intervention modalities may also be explored and tested, and long-term side effects of any should be noted. Comparisons between the different preventive methods must be made to conclude the intervention method and dose for recommendations for future preventive care. These recommendations can be used in the long term and offered in high-risk populations to reduce the incidence of ECC.

To better assess the real preventive effect of the experimental variable being tested, long term studies will assess the efficacy of the prevention modalities within the ages where there is a peak incidence of ECC. Preventive methods that will effectively and significantly reduce ECC levels within the time of highest incidence can be utilized in the future as an effective prenatal preventive care.

## Conclusion

Caries-preventive interventions applied to expectant mothers might result in reduced *S. mutans* levels in their children. Regarding caries levels the results were contradictory. Further studies are warranted to provide additional evidence.

## Supplementary Information

Below is the link to the electronic supplementary material.

Supplementary file1 (PDF 117 KB)

## Data Availability

The review data are included in the primary studies eligible of inclusion in the present systematic review.

## References

[CR1] Agili AI. A systematic review of population-based dental caries studies among children in Saudi Arabia Production and hosting by Elsevier. Saudi Dent J. 2013;25:3–11. 10.1016/j.sdentj.2012.10.002. 23960549 10.1016/j.sdentj.2012.10.002 PMC3723279

[CR2] Alantali K, Al-Halabi M, Hussein I, El-Tatari A, Hassan A, Kowash M. Changes in preschool children’s oral health-related quality of life following restorative dental general anaesthesia. Br Dent J. 2020;229:10. 10.1038/s41415-020-2335-7. 10.1038/s41415-020-2335-7 33247261

[CR3] Aljarallah FA, Alghanim HZ, Alanazi ABT. Prevalence of early childhood caries. Egyp J Hosp Med. 2018;70:8.

[CR4] American Academy of Pediatric Dentistry (AAPD) (2012) Guideline on infant oral health care. 35(6):13–4

[CR5] American Academy of Pediatric Dentistry (2024). Policy on social determinants of children’s oral health and health disparities. The Reference Manual of Pediatric Dentistry. Chicago, Ill.: American Academy of Pediatric Dentistry; 48–52

[CR6] Anil S, Anand PS. Early childhood caries: prevalence, risk factors, and prevention. Front Pediatr. 2017. 10.3389/fped.2017.00157. 28770188 10.3389/fped.2017.00157 PMC5514393

[CR7] Assiry AA. Transmission and colonization of Streptococcus mutans in children. Rev Med Microbiol. 2018;29:3. 10.1097/MRM.0000000000000140.

[CR8] Bahardoust M, Salari S, Ghotbi N, Rahimpour E, Haghmoradi M, Alipour H, Soleimani M. Association between prenatal vitamin D deficiency with dental caries in infants and children: a systematic review and meta-analysis. BMC Pregnancy Childbirth. 2024;24:256. 10.1186/s12884-024-06477-0. 38589811 10.1186/s12884-024-06477-0 PMC11000361

[CR9] Begzati A, Berisha M, Mrasori S, Xhemajli-Latifi B, Prokshi R, Haliti F, et al. (2015) Early Childhood Caries (ECC): Etiology, Clinical Consequences and Prevention. Emerging Trends in Oral Health Sciences and Dentistry. pp.31–56

[CR10] Beller EM, Glasziou PP, Altman DG, Hopewell S, Bastian H, Chalmers I, et al. PRISMA for abstracts: reporting systematic reviews in journal and conference abstracts. PLoS Med. 2013;10:4. 10.1371/journal.pmed.1001419. 10.1371/journal.pmed.1001419 PMC3621753 23585737

[CR11] Bergel E, Gibbons L, Rasines MG, Luetich A, Belizán JM. Maternal calcium supplementation during pregnancy and dental caries of children at 12 years of age: Follow-up of a randomized controlled trial. Acta Obstetr Gynecol Scand. 2010;89:11. 10.3109/00016349.2010.518228. 10.3109/00016349.2010.518228 20831450

[CR12] Birungi N, Fadnes LT, Okullo I, Kasangaki A, Nankabirwa V, Ndeezi G, et al. Effect of breastfeeding promotion on early childhood caries and breastfeeding duration among 5 year old children in Eastern Uganda: a cluster randomized trial. PLoS ONE. 2015;10:5. 10.1371/journal.pone.0125352. 10.1371/journal.pone.0125352 PMC4418833 25938681

[CR13] Borenstein M, Hedges LV, Higgins JPT, Rothstein HR. Introduction to Meta-Analysis. Chichester, UK: Wiley; 2009.

[CR14] Brambilla E, Felloni A, Gagliani M, Malerba A, García-Godoy F, Strohmenger L. Caries prevention during pregnancy: results of a 30-month study. J Am Dent Assoc. 1998;129:7. 10.14219/jada.archive.1998.0351 9685762

[CR15] Caufield PW, Cutter GR, Dasanayake AP. Initial acquisition of mutans streptococci by infants: evidence for a discrete window of infectivity. J Dent Res. 1993;72:1. 10.1177/00220345930720010501. 10.1177/00220345930720010501 8418105

[CR16] Çolak H, Dülgergil Ç, Dalli M, Hamidi M. Early childhood caries update: a review of causes, diagnoses, and treatments. J Nat Sci Biol Med. 2013. 10.4103/0976-9668.107257. 23633832 10.4103/0976-9668.107257 PMC3633299

[CR17] DerSimonian R, Laird N. Meta-analysis in clinical trials. Control Clin Trials. 1986;7:177–88. 3802833 10.1016/0197-2456(86)90046-2

[CR18] Drury TF, Horowitz AM, Ismail AI, Maertens MP, Rozier RG, Selwitz RH. Diagnosing and reporting early childhood caries for research purposes. J Public Health Dent Am Assoc Public Health Dent. 1999;59(3):192–7. 10.1111/j.1752-7325.1999.tb03268.x. 10.1111/j.1752-7325.1999.tb03268.x 10649591

[CR19] Ezer M, Swoboda N, Farkouh D (2010). Early Childhood Caries: The Dental Disease of Infants - Oral Health Group. Available from: https://www.oralhealthgroup.com/features/early-childhood-caries-the-dental-disease-of-infants/ Accessed: 3 April 2022

[CR21] Folayan MO, el Tantawi M, Aly NM, Al-Batayneh OB, Schroth RJ, Castillo JL, et al. Association between early childhood caries and poverty in low and middle income countries. BMC Oral Health. 2020. 10.1186/s12903-019-0997-9. 31906944 10.1186/s12903-019-0997-9 PMC6945445

[CR22] Folayan MO, Coelho EM, Ayouni I, et al. Association between early childhood caries and parental education and the link to the sustainable development goal 4: a scoping review. BMC Oral Health. 2024;24:517. 10.1186/s12903-024-04291-w. 38698356 10.1186/s12903-024-04291-w PMC11064360

[CR23] Garcia MB, Nör JE, Schneider LG, Bretz WA. A model for clinical evaluation of the effect of antimicrobial agents on carious dentin. Am J Dent. 2001;14:3. 11572285

[CR24] Guyatt GH, Oxman AD, Schünemann HJ, Tugwell P, Knottnerus A. GRADE guidelines: a new series of articles in the journal of clinical epidemiology. J Clin Epidemiol. 2011;64:380–2. 21185693 10.1016/j.jclinepi.2010.09.011

[CR25] Harris R, Nicoll AD, Adair PM, Pine CM. Risk factors for dental caries in young children: a systematic review of the literature. Community Dent Health. 2004;21:71–85. 15072476

[CR26] Harrison RL, Veronneau J, Leroux B. Effectiveness of maternal counseling in reducing caries in cree children. J Dent Res. 2012. 10.1177/0022034512459758. 22983408 10.1177/0022034512459758

[CR27] Higgins JPT, Thomas J, Chandler J, Cumpston M, Li T, Page MJ, Welch VA (editors). Cochrane Handbook for Systematic Reviews of Interventions version 6.5 (updated August 2024). Cochrane, 2024. Available from www.training.cochrane.org/handbook. Accessed: 5 December 2024

[CR28] Holmes R, Porter J, Vernazza C, Tsakos G, Ryan R, Dennes M. Children’s Dental Health Survey 2013 Country specific report: England. 2015. https://webarchive.nationalarchives.gov.uk/ukgwa/20171010184810tf_/http://content.digital.nhs.uk/catalogue/PUB17137/CDHS2013-England-Report.pdf Accessed: 3 April 2022

[CR29] Ioannidis JP. Interpretation of tests of heterogeneity and bias in meta-analysis. J Eval Clin Pract. 2008;14:951–7. 19018930 10.1111/j.1365-2753.2008.00986.x

[CR30] Ismail AI. Prevention of early childhood caries. Comm Dent Oral Epidemiol. 1998. 10.1111/j.1600-0528.1998.tb02094.x. 10.1111/j.1600-0528.1998.tb02094.x 9671200

[CR31] Köhler B, Bratthall D, Krasse B. Preventive measures in mothers influence the establishment of the bacterium Streptococcus mutans in their infants. Arch Oral Biol. 1983. 10.1016/0003-9969(83)90151-6. 6574733 10.1016/0003-9969(83)90151-6

[CR32] Kowash MB. Severity of early childhood caries in preschool children attending Al-Ain Dental Centre. United Arab Emirates: European Archives of Paediatric Dentistry; 2015. 10.1007/s40368-014-0164-6. 10.1007/s40368-014-0164-6 25526933

[CR33] Kowash MB, Alkhabuli JO, Dafaalla SA, Shah A, Khamis AH. Early childhood caries and associated risk factors among preschool children in Ras Al-Khaimah. United Arab Emirates: European Archives of Paediatric Dentistry; 2017. 10.1007/s40368-017-0278-8. 10.1007/s40368-017-0278-8 28243836

[CR34] Lee C, Rezaiamira N, Jeffcott E, Oberg D, Domoto P, Weinstein P. Teaching parents at WIC clinics to examine their high caries-risk babies. ASDC J Dent Child. 1994;61:5–6. 7897004

[CR35] Leverett DH, Adair SM, Vaughan BW, Proskin HM, Moss ME. Randomized clinical trial of the effect of prenatal fluoride supplements in preventing dental caries. Car Res. 1997. 10.1159/000262394. 10.1159/000262394 9165186

[CR36] Lucey SM. Oral health promotion initiated during pregnancy successful in reducing early childhood caries. Evid-Based Dent. 2009. 10.1038/sj.ebd.6400677. 20023610 10.1038/sj.ebd.6400677

[CR37] MacHiulskiene V, Campus G, Carvalho JC, Dige I, Ekstrand KR, Jablonski-Momeni A, et al. Terminology of dental caries and dental caries management: consensus report of a workshop organized by ORCA and cariology research group of IADR. Caries Res. 2020;54(1):7–14. 10.1159/000503309. 31590168 10.1159/000503309

[CR38] Marriott BP, White AJ, Hadden L, Davies JC, Wallingford JC. How well are infant and young child World Health Organization (WHO) feeding indicators associated with growth outcomes? An example from Cambodia: Maternal and Child Nutrition; 2010. 10.1111/j.1740-8709.2009.00217.x. 10.1111/j.1740-8709.2009.00217.x PMC6860499 21050390

[CR39] Mattheus D (2015). Oral Health Outcomes for Children in Hawaii: Not Much to Smile About. J Dent Prob Solut 10.17352/2394-8418.000014

[CR40] Nakai Y, Shinga-Ishihara C, Kaji M, Moriya K, Murakami-Yamanaka K, Takimura M. Xylitol gum and maternal transmission of mutans streptococci. J Dent Res. 2010. 10.1177/0022034509352958. 19948944 10.1177/0022034509352958

[CR41] Nørrisgaard PE, Haubek D, Kühnisch J, Chawes BL, Stokholm J, Bønnelykke K, Bisgaard H. Association of high-dose vitamin D supplementation during pregnancy with the risk of enamel defects in offspring: a 6-year follow-up of a randomized clinical trial. JAMA Pediatr. 2019;173(10):924–30. 10.1001/jamapediatrics.2019.2545. 31381020 10.1001/jamapediatrics.2019.2545 PMC6686764

[CR42] Page MJ, McKenzie JE, Bossuyt PM, Boutron I, Hoffmann TC, Mulrow CD, et al. Updating guidance for reporting systematic reviews: development of the PRISMA 2020 statement. J Clin Epidemiol. 2021. 10.1016/j.jclinepi.2021.02.003. 33577987 10.1016/j.jclinepi.2021.02.003

[CR43] Plutzer K. Dealing with missing outcomes: lessons from a randomized trial of a prenatal intervention to prevent early childhood caries. Open Dentis J. 2010. 10.2174/1874210601004020055. 10.2174/1874210601004020055 PMC2944987 20871748

[CR44] Plutzer K, Spencer AJ. Efficacy of an oral health promotion intervention in the prevention of early childhood caries. Comm Dentis Oral Epidemiol. 2008. 10.1111/j.1600-0528.2007.00414.x. 10.1111/j.1600-0528.2007.00414.x 19145720

[CR45] Plutzer K, John Spencer A, Keirse MJNC. Reassessment at 6–7 years of age of a randomized controlled trial initiated before birth to prevent early childhood caries. Comm Dentis Oral Epidemiol. 2012. 10.1111/j.1600-0528.2011.00643.x. 10.1111/j.1600-0528.2011.00643.x 22022927

[CR46] Reisine S, Tellez M, Willem J, Sohn W, Ismail A. Relationship between caregiver’s and child’s caries prevalence among disadvantaged African Americans. Commun Dent Oral Epidemiol. 2008;36(3):191–200. 10.1111/j.1600-0528.2007.00392.x 18474051

[CR47] Saxena V, Datla A, Deheriya A, Shoukath S, Tiwari N, Bhargava A. Maternal oral health education for predicting early childhood caries among preschool children: a systematic review and meta-analysis. J Clin Diagn Res. 2024;18(5):42–8.

[CR48] Schroth RJ, Lavelle C, Tate R, Bruce S, Billings RJ, Moffatt MEK. Prenatal vitamin D and dental caries in infants. Pediatrics. 2014. 10.1542/peds.2013-2215. 24753535 10.1542/peds.2013-2215

[CR49] Shamseer L, Moher D, Clarke M, Ghersi D, Liberati A, Petticrew M, et al. Preferred reporting items for systematic review and meta-analysis protocols (prisma-p) 2015: Elaboration and explanation. BMJ: BMJ Publishing Group; 2015. 10.1136/bmj.g7647. 10.1136/bmj.g7647 25555855

[CR50] Tinanoff N, O’sullivan DM. Early childhood caries: overview and recent findings. Am Acad Pediat Dent. 1997;19(1):12–6. 9048407

[CR51] Weintraub JA. Prevention of early childhood caries: a public health perspective. Commun Dent Oral Epidemiol. 1998. 10.1111/j.1600-0528.1998.tb02095.x. 10.1111/j.1600-0528.1998.tb02095.x 9671201

[CR52] Wyne AH. Early childhood caries: Nomenclature and case definition. Commun Dent Oral Epidemiol. 1999. 10.1111/j.1600-0528.1999.tb02026.x. 10.1111/j.1600-0528.1999.tb02026.x 10503790

[CR53] Xiao J, Alkhers N, Kopycka-Kedzierawski DT, Billings RJ, Wu TT, Castillo DA, et al. Prenatal oral health care and early childhood caries prevention: a systematic review and meta-analysis. Caries Res. 2019. 10.1159/000495187. 30630167 10.1159/000495187 PMC6554051

[CR54] Zanata RL, de Navarro MFL, Pereira JC, Franco EB, Lauris JRP, Barbosa SH. Effect of caries preventive measures directed to expectant mothers on caries experience in their children. Braz Dent J. 2003. 10.1590/S0103-64402003000200001. 12964648 10.1590/s0103-64402003000200001

