# Supplementary material for: Do clinical caries-preventive interventions applied to expectant mothers affect caries-related parameters in their children? A systematic review
Source: Eur Arch Paediatr Dent. 2025 Apr 9;26(4):633–44. doi: 10.1007/s40368-025-01025-6 (PMC12283861; doi:10.1007/s40368-025-01025-6)
Supplement: Supplementary file 1 — Supplementary file1 (PDF 117 KB) [file 40368_2025_1025_MOESM1_ESM.pdf]

Do clinical caries-preventive interventions applied to expectant mothers affect caries-related parameters in their children? A systematic review [Supplementary material]

Supplementary Table 1. Eligibility criteria

Domain	Inclusion criteria	Exclusion criteria
**Participants**	▪ Pregnant individuals of any age.	▪ Individuals with medical conditions.
**Interventions**	▪ Caries preventive measures involving the supplementation of chemical agents like fluoride or xylitol, the application of antimicrobial agents, etc.	▪ Studies involving the educational or behavioural interventions. Interventions directed to both pregnant individuals and their children following birth.
**Comparisons**	▪ Placebo, other interventions, standard intervention or no intervention.	
**Outcomes**	▪ Primarily, outcomes reflecting caries experience in their children of any age (both genders), like DMFT, DMFS, dmft, dmfs, percentage free of caries, etc. or microbiological parameters related to caries, like *Streptococcus mutans* levels, etc..<br>▪ Secondarily, patient reported outcomes and socioeconomical assessments.<br>Where needed, numerical data where transformed into the desired formats and tested statistically using MedCalc (2016 MedCalc, Belgium) and QuickCalcs (2016 GraphPad Software, Inc, USA).	
**Study design**	▪ Randomized clinical trials.	▪ Non-randomized studies.<br>▪ Non-comparative studies (case reports and case series).<br>▪ Animal studies.<br>▪ Ex vivo, in vitro, in silico studies.<br>▪ Reviews (traditional reviews, systematic reviews and meta-analyses).

Supplementary Table 2. Strategy for database search [until November 17th, 2024].

Database [2024 11 17]	Search strategy	Hits
**PubMed**	((“randomized controlled trial”[pt] OR “controlled clinical trial”[pt] OR randomized[tiab] OR placebo[tiab] OR randomly[tiab]) NOT (animals[mh] NOT humans[mh])) AND (gingival[tiab] OR gingivitis[tiab] OR bleeding[tiab] OR periodontal[tiab] OR periodontitis[tiab] OR plaque[tiab] OR “oral flora”[tiab] OR biofilm[tiab] OR biofilms[tiab] OR decalcification[tiab] OR decalcifications[tiab] OR caries[tiab] OR carious[tiab] OR “white spot”[tiab] OR “white spots”[tiab] OR WSL[tiab] OR “oral health” [tiab] OR “oral hygiene” [tiab]) AND (pregnancy[tiab] OR pregnant[tiab] OR gestation[tiab] OR gravidity[tiab] OR impregnation[tiab] OR anticipating[tiab] OR expecting[tiab] OR fecundity[tiab] OR conception[tiab] OR procreation[tiab] OR reproduction[tiab] OR parturiency[tiab])	**1667**
**Cochrane Central Register of Controlled Trials**	(gingival OR gingivitis OR bleeding OR periodontal OR periodontitis OR plaque OR "oral flora" OR biofilm OR biofilms OR decalcification OR decalcifications OR caries OR carious OR "white spot" OR "white spots" OR WSL OR "oral health" OR "oral hygiene") AND (pregnancy OR pregnant OR gestation OR gravidity OR impregnation OR anticipating OR expecting OR fecundity OR conception OR procreation OR reproduction OR parturiency) in Record Title - (Word variations have been searched)	**317**
**Cochrane Database of Systematic Reviews**	(gingival OR gingivitis OR bleeding OR periodontal OR periodontitis OR plaque OR "oral flora" OR biofilm OR biofilms OR decalcification OR decalcifications OR caries OR carious OR "white spot" OR "white spots" OR WSL OR "oral health" OR "oral hygiene") AND (pregnancy OR pregnant OR gestation OR gravidity OR impregnation OR anticipating OR expecting OR fecundity OR conception OR procreation OR reproduction OR parturiency) in Record Title - (Word variations have been searched)	**4**
**Scopus**	TITLE-ABS(("randomized controlled trial" OR "controlled clinical trial" OR randomized OR placebo OR randomly) AND (gingival OR gingivitis OR bleeding OR periodontal OR periodontitis OR plaque OR "oral flora" OR biofilm OR biofilms OR decalcification OR decalcifications OR caries OR carious OR "white spot" OR "white spots" OR wsl OR "oral health" OR "oral hygiene") AND (pregnancy OR pregnant OR gestation OR gravidity OR impregnation OR anticipating OR expecting OR fecundity OR conception OR procreation OR reproduction OR parturiency)) AND (LIMIT-TO (SUBJAREA,"DENT")) AND (LIMIT-TO (EXACTKEYWORD,"Randomized Controlled Trial"))	**81**
**Web of Science™**	gingival OR gingivitis OR bleeding OR periodontal OR periodontitis OR plaque OR "oral flora" OR biofilm OR biofilms OR decalcification OR decalcifications OR caries OR carious OR "white spot" OR "white spots" OR WSL OR "oral health" OR "oral hygiene") AND (pregnancy OR pregnant OR gestation OR gravidity OR impregnation OR anticipating OR expecting OR fecundity OR conception OR procreation OR reproduction OR parturiency) (Title) and Preprint Citation Index (Exclude – Database) and Dentistry Oral Surgery Medicine (Research Areas) and Randomized Controlled Trials As Topic (MeSH Headings)<br>Timespan: All years. All Databases; Search language=Auto	**21**
**ProQuest Dissertations and Theses Global**	title((gingival OR gingivitis OR bleeding OR periodontal OR periodontitis OR plaque OR "oral flora" OR biofilm OR biofilms OR decalcification OR decalcifications OR caries OR carious OR "white spot" OR "white spots" OR WSL OR "oral health" OR "oral hygiene") AND (pregnancy OR pregnant OR gestation OR gravidity OR impregnation OR anticipating OR expecting OR fecundity OR conception OR procreation OR reproduction OR parturiency)) in Full Text	**54**
**Google Scholar**	allintitle: randomized caries pregnancy gingival gingivitis bleeding periodontal periodontitis plaque oral flora biofilm biofilms decalcification decalcifications caries carious "white spot" "white spots" WSL "oral health" "oral hygiene"pregnancy pregnant gestation gravidity impregnation anticipating expecting fecundity conception procreation reproduction parturiency	**5**
